# Increasing River Temperature Shifts Impact the Yangtze Ecosystem: Evidence from the Endangered Chinese Sturgeon

**DOI:** 10.3390/ani9080583

**Published:** 2019-08-20

**Authors:** Hui Zhang, Myounghee Kang, Jinming Wu, Chengyou Wang, Junyi Li, Hao Du, Haile Yang, Qiwei Wei

**Affiliations:** 1 Key Laboratory of Freshwater Biodiversity Conservation, Ministry of Agriculture and Rural Affairs of P. R. China, Wuhan 430223, Hubei Province, China; 2 Yangtze River Fisheries Research Institute, Chinese Academy of Fishery Sciences, Wuhan 430223, Hubei Province, China; 3 Department of Maritime Police and Production System, The Institute of Marine Industry, Gyeongsang National University, Tongyeong-si 53064, Gyeongsangnam-do, Korea

**Keywords:** river thermal regime, cascade dam development, *Acipenser sinensis*, spawning time, gonad development, Yangtze River, China

## Abstract

**Simple Summary:**

A river thermal regime is critically influencing the aquatic ecosystem and human-induced water temperature alteration occurs commonly worldwide. In large river systems, thermal alterations do not readily arise because of the huge water volume. The Yangtze River has the third greatest water flow, is the third longest river, and is one of the most human-influenced rivers in the world. Here, we found that the entire water temperature regime in the Yangtze River has been changed by damming and will shift further under future dam construction. We think that this, in conjunction with the impacts of global warming, means that saving the Chinese sturgeon by regulating water temperature is critically difficult. Also, river thermal shifts not only threaten fish but also affect the whole Yangtze aquatic ecosystem.

**Abstract:**

The Yangtze River has the third greatest water flow and is one of the most human-influenced rivers in the world. Since 1950, this river system has experienced drastic human interventions, leading to various environmental changes, including water temperature. In this study, based on observations during the past sixty years, we found that the seasonal temperature regime has been altered, both temporally (1–5 °C variation) and spatially (>626 km distance). Temperature shifts not only delay the timing of fish spawning directly, but also lead to degeneration in gonad development. Temperature regime alterations have delayed the suitable spawning temperature window by approximately 29 days over a decade (2003–2016). It confirmed that a period of lower temperature, higher cumulative temperature, and relatively higher temperature differences promoted the maturation of potential spawners based on the correlation analysis (*p* < 0.05). Also, thermal alterations were highly correlated with reservoir capacity upstream (R^2^ = 0.866). On-going cascade dam construction and global warming will lead to further temperature shifts. Currently, rigorous protection measures on the breeding population of the Chinese sturgeon and its critical habitats is urgently needed to prevent the crisis of the species extinction. Increasing river thermal shifts not only threaten the Chinese sturgeon but also affect the entire Yangtze aquatic ecosystem.

## 1. Introduction

The adverse effects of altered river temperature on aquatic organisms and ecosystem structure and function have been widely reviewed [1,2,3]. In large river systems, however, long-term and large-scale thermal alterations and their consequences on aquatic ecosystems have been little studied. The Yangtze River has the third greatest water flow, is the third longest river, and is one of the most human-influenced rivers in the world [4]. The great Yangtze River is essential both for freshwater fisheries development and aquatic biodiversity conservation in China [5,6,7,8]. However, this river system has experienced drastic human interventions (e.g., damming) since 1950, resulting in a number of environmental changes, including water temperature [9].

The Chinese sturgeon, *Acipenser sinensis,* is a large anadromous fish species, which has been historically distributed in the West Pacific and most of the East Asian river systems [10]. In recent decades, the species has been appearing mainly in the continental shelf of the East China Sea and the main stem of the Yangtze River [11]. Since the 1970s, the natural population of the species began to extremely decline because of habitat degradation and over-fishing. The migration route of the species toward upstream spawning sites has been blocked by the Gezhouba Dam (without any fish passage) in the Yangtze River since 1981 [11,12]. In the past 30 years, the natural reproduction of this species can be found only in just below the Gezhouba hydrodam, rendering upstream spawning sites along the 600 km river stretch inaccessible to spawners [13,14]. In 1989, the species was listed as a top-level protected animal in China, and in 2009, it was listed as a Critically Endangered species in the Red List of the International Union for Conservation of Nature.

Since the 2000s, it was confirmed that the only remaining spawning area of the Chinese sturgeon just below the Gezhouba Dam was very constrained for the dwindling spawner cohort that still spawned at the site [13,15]. Only 200 spawning adults entered annually into the spawning reach from the sea by hydro-acoustic assessment research [16,17], and unfortunately, only tens of adults spawned successfully [12]. On the other hand, more than 90% of the juvenile Chinese sturgeon in the Yangtze estuary originated from natural reproduction which was confirmed by mark-recapture and molecular genetic identification tests, although the controlled propagation and augmentation have been conducted as early as 1983 [18,19]. However, the spawning activity of the fish was fully interrupted in recent years [20,21], which means that the wild population of the fish is very close to extinction. Thus, it is of critical importance to analyze the factors influencing the pre-spawning conditions that determine and trigger spawning activities of adults in the river.

It is well known that temperate fish species are annual spawners and mainly rely on annually cycling cues (such as temperature) to synchronize their reproductive cycle [22]. In the Yangtze River, controlling water temperature was recommended as a means of maintaining the spawning activity of the Chinese sturgeon and saving the fish from near extinction [23,24,25]. In this study, we compared the river temperature regimes in the only remaining spawning area of the Chinese sturgeon from 1981 to 2016. The possible effects of thermal alteration on gonad development and spawning timing of the fish were examined. We further analyzed the trends of future river temperature regime. This study aimed to reveal the alterations (shifts) in the temperature regime, and its effects on the timing and gonadal development level of the Chinese sturgeon to find the possible mitigation measures. The study can provide important information on developing conservation strategies under man-induced and climate changes not only for the Chinese sturgeon but also for other fish species, hopefully for the entire aquatic ecosystem of the Yangtze River basin.

## 2. Materials and Methods

### 2.1. Study Area

**Figure 1:**
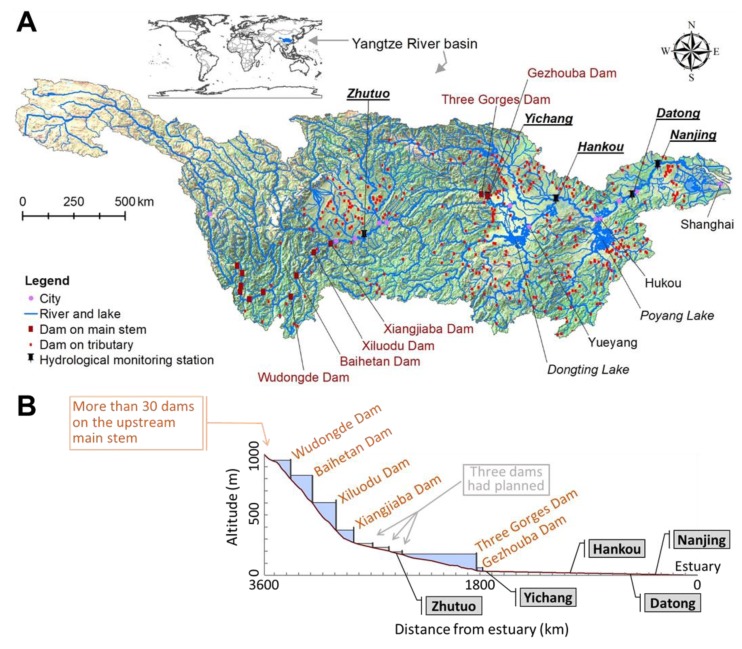
Study area of the Yangtze Basin. (**A**) Dams and five hydrological monitoring stations in the Yangtze main stem (Zhutuo, river km (rkm) 2474.2, rkm 0 is at the Yangtze estuary, Yichang, rkm 1669.2, Hankou, rkm 1043.2, Datong, rkm 553.9, and Nanjing, rkm 348.1). (**B**) Dams, hydrological monitoring stations, and longitudinal profile along the river.

The Yangtze River is the largest river in Asia (Figure 1), it is more than 6300 km in length and has a catchment area of 1.8 × 10^6^ km^2^. It has 437 tributaries with catchment areas greater than 1000 km^2^, and 27 lakes larger than 100 km^2^ [5,6,7]. The geographic range of the freshwater life history of the Chinese sturgeon has been very large, that is a stretch of approximately 3000 km of the Yangtze River main stem [10,11]. From the upstream to the downstream reaches, this stretch of the river can be divided into three segments:

(1) Upper section: The upper reaches above Yichang City (approximately 1300 km in riverine length), includes the Lower Jinsha River and the Upper Yangtze River, flowing through mountainous areas with about 500 m elevation. The river shorelines are uneven with many central bars, point bars, and reefs in the meandering riverbed. The river width ranges from approximately 200–300 m to 600–800 m depending on various types of gorges. The maximum river width can reach up to 1500–2000 m at certain points. The average river thalweg depth is approximately 12 m with the range of 4–43 m. The riverbed gradient is 2.7 × 10^−4^–4.0 × 10^−4^. During the study period, the annual flow discharge at Pingshan (~30 km above the Xiangjiaba Dam) was 4540 m^3^/s, and the annual sediment content in the water averaged 1.77 kg/m^3^. The average velocity over depth at the river thalweg was 1.7 m/s with the range between 0.5 and 3.3 m/s. More than 16 historic spawning areas of the Chinese sturgeon were reported in this reach [10,11].

(2) Middle section: The Middle Reach of the Yangtze River expands from Yichang to Hukou (approximately 900 km in length). This is mainly an alluvial river section flowing through the plains. The river has braided and meander channels with a river width commonly wider than 1000 m having mainly sandy substrate. The riverbed gradient is 0.097 × 10^−4^–0.203 × 10^−4^. The annual flow discharge at Yichang reached 13,900 m^3^/s, and the sediment content in the water averaged 1.14 kg/m^3^. The average flow velocity throughout the whole reach was approximately 2.0–3.0 m/s and 1.0–1.6 m/s during flood and dry seasons, respectively. The only known recent spawning area in this entire stretch over the past 32 years (1981–2012) was documented to be right below the Gezhouba Dam [11,13,14,15].

(3) Lower section: The Lower Reach of the Yangtze River is from Hukou to the Yangtze River estuary (approximately 800 km in riverine length) and is mainly braided by a channel with the river width of larger than 2 km. The riverbed gradient is 0.193 × 10^−4^–0.579 × 10^−4^. The annual flow discharge around the Tongling (~7 km below Datong) reach was 28,700 m^3^/s, and the annual sediment contents in the water averaged around 0.486 kg/m^3^.

The river is essential for freshwater fisheries development and aquatic biodiversity conservation in China [5,6,7]. Its fishery capture yields represented 60% of Chinese inland capture production in the 1950s, and currently the basin production (including capture and aquaculture) still constitutes 60% of Chinese inland fishery production. Of the 35 major freshwater aquaculture species in China, 26 are from the Yangtze Basin. There are also approximately 416 fish species (subspecies) in the Yangtze River basin, of which 178 are endemic [8]. Currently, a total of 70 aquatic animals in the Yangtze are listed as internationally and/or nationally protected species, including the well-known Baiji *Lipotes vexillifer*, Yangtze finless porpoise *Neophocaena asiaeorientalis*, Chinese paddlefish *Psephurus gladius*, and Chinese sturgeon *A. sinensis* [5,6,7]. However, since the 1950s, this river system has experienced increasingly drastic human interventions (e.g., damming), which have been greatly threatening the entire Yangtze aquatic ecosystem [5,6,7].

### 2.2. Water Temperature Measurement

The water temperature data of four stations (Yichang, data period 1956–2016, Hankou, 1995–2014, Datong, 1995–2014, and Nanjing, 2005–2014) below the Gezhouba Dam (Figure 1) were obtained from the *Changjiang (Yangtze) Hydrological Yearbook*. The yearbooks were issued by the Bureau of Hydrology, Changjiang Water Resources Commission, which is the only official agency to observe the hydrological elements in the Yangtze Basin. The water temperature at each station was measured by a mercury thermometer at 08:00 am every day, and this temperature was regarded as the daily baseline water temperature.

At Yichang station during 2015–2016 (Figure 1), the water temperature (at 1 m depth) was measured by a HOBO Pendant^®^ Temperature/Light 64K Data Logger (accuracy ±0.53 °C and resolution 0.14 °C, Onset Computer Corporation, Bourne, MA, USA) every hour. The mean of 24 measured values in each day was used as the daily water temperature.

### 2.3. Assessing the Maturation Process of Spawners

From 1982 to 1994, spawning Chinese sturgeons were captured by fishermen annually in autumn (from the end of September to early November) [11,26]. Sampling locations were an overall river stretch of approximately 30 km below the Gezhouba Dam. Since 1995, however, the sampling has only been undertaken along a river stretch of approximately 3–5 km below the dam since the area was identified as the only remaining spawning section. Capture tools were mainly setlines and sweeping trammel nets. The setline was the main tool after 1991. Fish were assessed for sex and maturity stage at the river bank immediately after capture. Thereafter, some specimens were released or some were taken for further examination by transporting to the hatchery. The fish catches varied annually between 30 and 50 specimens from 1982 to 1998, and declined to no more than 10 to 30 fish annually in between 1999 and 2003, except for 2002.

Two indicators were used to describe the maturation status of the breeding population of the Chinese sturgeon below the Gezhouba Dam. The indicators are the percentage of the degeneration of gonadal tissue from the observed cohorts and the percentage of the maturation in gonad. The percentage of degeneration was defined as the ratio of adult females showing regressing or degenerating ovaries [11], in which a gonadosomatic index (GSI = gonadal weight/body weight) and an egg diameter do not reach stage IV (last stage before full ripeness ready to spawn), however the gonadal sections showed that the yolk was being re-absorbed. The information on the degeneration percentage of the breeding populations in between 1984 and 1993 was directly taken from Wei et al. [11], which described that the degeneration values decreased since 1984, yet varied from year to year with the mean of 41.8% ± 30.5% (range 0–80.0%). The percentage of the maturation was defined as the ratio of mature individuals (adults in stage IV–V of the ovulation stage) that can be used to conduct controlled propagation [26]. The data for the maturation percentage used in this study were in the period 1982–2003 (except 2002) and were directly acquired from the published source by Xiao et al. [26]. Results showed that maturation rates were low in 1982 and 1983 (13.9% and 15.6%), but rose conspicuously between 1984 and 1990, except 1989, with fluctuating rates between 48.4% and 58.0% (50.1% ± 9.9%). These rates increased further and fluctuated between 69.2% and 96.7% in the period of 1991–2002. In 2003, the maturation rate reached 100%. Extensive details on degeneration and maturation rates have been presented in the publications by Wei et al. [11] and Xiao et al [26], respectively.

Since 2009, it is not permitted to capture the Chinese sturgeon even for scientific research purposes. Therefore, only several occasionally by-catch individuals can be used to ascertain the gonad development status of the adults. Here, a by-catch specimen was found below and near the Gezhouba Dam on 25 November 2014.

### 2.4. Description of the Thermal Regime

A total of six indicators were developed to describe the temperature regime (Table 1). These indicators were selected mainly based on two considerations: (1) the present state of knowledge on the effects of the water temperature regime on the course of sturgeon maturation, precisely speaking, how the Chinese sturgeon sustains in a low range of water temperature which affects the completion of the ovarian cycle [27]. (2) The indicators are widely used to describe hydrological alteration by the nature conservancy [28]. The indicators were defined and calculated for one year of water temperature, that is from November 1 to October 31 of the following year. This period was chosen because spawning activities of the Chinese sturgeon began to occur around November 1 [13,29], in which was the time when a present spawning cohort (adults in stage IV) completed spawning and a next spawning cohort (adults in stage III) entered into a key period for gonad development.

### 2.5. Temperature Suitability Evaluation

This suitability index can be used as a standard index to evaluate the daily water temperature for spawning fish. The temperature suitability index for spawning of the Chinese sturgeon was imported from Zhang et al. [30]. It was based on ten spawning temperatures in historic spawning areas [10] and 20 spawning temperatures in recent spawning areas [29]. A frequency statistic was first conducted using a 1 °C temperature scale. The temperature range with the highest frequency of runs was regarded as suitability index 1, and thereafter an index of other temperature ranges was obtained by calculating the ratio of its frequency to the highest frequency. The suitability index for the Chinese sturgeon spawning at 17–18 °C, 18–19 °C, 19–20 °C, and 20–21 °C was 0.6, 1, 1, and 0.4, respectively. In addition, the temperature suitability index for the four major Chinese carps (FMCC; i.e., black carp *Mylopharyngodon piceus*, grass carp *Ctenopharyngodon idella*, silver carp *Hypophthalmichthys molitrix*, and bighead carp *H. nobilis*) was imported from Wang et al. [31], which was itself based on previous studies of Yi et al. [32]. When the water temperature increased to 15 °C (minimum temperature for adult carp gonad development), the suitability index was defined as 0.5. When the water temperature increased to 18 °C (minimum temperature for spawning of these carp), the suitability index was defined as 1. Finally, the suitability index of daily water temperature for FMCC gonad development and spawning, and Chinese sturgeon spawning in 1995–2014, was evaluated by Visual Basic for Applications in Microsoft^®^ Excel^®^ 2016.

### 2.6. Estimating Reservoir Capacity

The data of reservoir capacity were from the *Yangtze River Yearbook* from 1993 to 2017. These yearbooks include the total reservoir capacity from 1992 to 2016 in the entire Yangtze Basin. We estimated the total reservoir capacity in the upper Yangtze (above Gezhouba Dam) by assuming that it took a fixed proportion (41.8%) of the whole Yangtze [33].

### 2.7. Statistical Analysis

Mann–Kendall trend tests (two-tailed) were used to test whether there was a variation trend in the average water temperatures monthly and yearly in the period 1956–2016 (61 years) at Yichang station (Figure 1) [30]. The null hypothesis, H_0_, for these tests is that there was no trend in the series. The three alternative hypotheses: that there was a negative, a non-null, or a positive trend could be chosen. The Mann–Kendall tests are based on the calculation of Kendall’s tau measure of association between two samples, which is itself based on the ranks within the samples. The calculation was implemented by a complete statistical add-in, XLSTAT 2018 (Addinsoft SARL, Paris, France) for Microsoft^®^ Excel^®^ 2016.

Data periods (Supplemental Table S1) were divided into three stages according to the operation status of the Three Gorges Dam (TGD): before the TGD (before 2002), during trial operation of the TGD (2003–2009), and after the TGD (after 2010). Seasons (spring to winter) were defined from March to February of the following year based on water temperature curves (rise and fall). Each month was divided into three sub-periods (early 10 days, middle 10 days, and later 8–11 days. For instance, April was divided into E-Apr, M-Apr, and L-Apr) to investigate the fine timescale changes. The regression analysis between total reservoir capacity in the upper Yangtze (TRC_uY_) and year, between annual water temperature in M-Apr, M-Dec, and TRC_uY_, were conducted in Microsoft^®^ Excel^®^ 2016. Exponential, linear, logarithmic, polynomial, and power analyses were tested, and the equation with the highest R^2^ and simplest expression was selected.

## 3. Results

### 3.1. River Temperature Shifts

**Figure 2:**
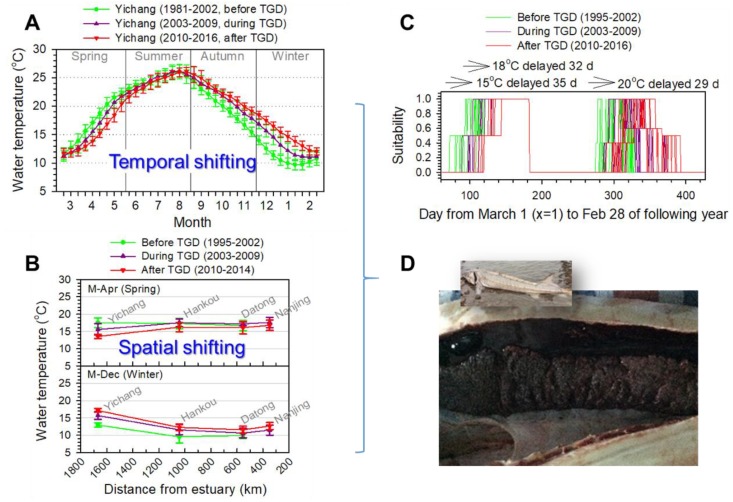
Increasing river temperature shifts threaten the spawning of Yangtze fish. (**A**) Temporal shift in water temperature at Yichang station (spawning area of the Chinese sturgeon) during 1981–2016. (**B**) Spatial shift of temperature deviations from Yichang (i.e., Gezhouba Dam, the lowermost dam on the main stem) to estuary (Supplemental Table S2). (**C**) Spawning window delay in spring for the four major Chinese carps and autumn for the Chinese sturgeon due to the shifting temperature regime. (**D**) Gonad development failure of the Chinese sturgeon observed in 2014, probably due to the altered temperature regime.

We found that water temperature curves had shifted in three stages of the Three Gorges Dam (TGD) (before, during, and after the TGD, Figure 2A,B). When comparing the ‘before’ and ‘after’ stages, we found that in spring, the water temperature decreased by ~2 °C, summer was generally the same with a less than 1 °C decrease. In autumn it increased by 1–3.5 °C, and in winter it increased by 2–5 °C. In the temperature profile along the river (rkm 1669.2–348.1) in M-Apr and M-Dec (Figure 2B), temperature alterations (increase or decrease) and varied temperature gradient from Yichang (i.e., Gezhouba Dam, the lowermost dam) to Nanjing (i.e., near the estuary) were clearly observed. In M-Dec at Hankou, the mean temperature increased 2.7 °C between the ‘before’ and ‘after’ TGD stages. The increase was beyond the 1 standard deviation range (1.8 °C) of the ‘before’ stage. This implies that the water temperature needs more than a 626-km reach to recover its natural status.

### 3.2. Maturation of Breeding Population in Relation to the Thermal Regime

The correlation analysis between the maturation percentage of the observed spawners and the temperature regime indicators showed significantly positive correlation in winter (December, January, February, March) and in October (Table 2). Low temperature at various time windows (1-day min, 3-day min, 7-day min, 30-day min, 90-day min), the cumulative temperature, and relative temperature differences revealed significantly positive correlation with the onset of final maturation and spawning activity. No significant correlation was found for other indicators such as monthly water temperature in Nov and April–September, high temperatures, and the time window for extreme temperature. The correlation analysis between the percentage of degenerating gonads and the temperature regime indicators showed a negative correlation in the 90-day min of low temperature and the cumulative temperature window, however no statistical difference was found in other indicators. Note that the 90-day min of low temperature and the cumulative temperature had a positive correlation in the maturation percentage of gonads yet a negative correlation in the percentage of degenerating gonads.

In summary, the gonad development status of the breeding population was correlated with water temperature in the winter and early spring (December to March), low temperature, cumulative temperature, and relative temperature difference. The indicators of low temperature, cumulative temperature, and relative temperature difference increased after the Three Gorges Reservoir which gradually reached full operational status during the period 2010–2016. The 7-day minimum temperature (11.55 °C) was close to the lowest temperature measured in the historic spawning area (11.61 °C). This fact should accordingly be beneficial for the gonadal development of the Chinese sturgeon. However, the alteration of temperatures during the winter and early spring (December to March) became very complicated, for instance, temperatures obviously increased in Dec (+4.67 °C), Jan (+4.57 °C), and Feb (+2.26 °C), while they decreased in Mar (−0.73 °C). The lowest temperature period, which usually occurred in Jan (10.07 °C) during the 1982–2001, was shifted to Mar (11.93 °C) in 2010–2016. Thus, this temperature shift occurred over approximately two months. This shift in the lowest temperature period would have adverse effects on gonad development of the Chinese sturgeon. For instance, the gonad development failure of the Chinese sturgeon was observed in a by-catch specimen below and near the Gezhouba Dam on 25 November 2014, during the spawning season (Figure 2D).

### 3.3. Spawning Delay with the Altered Temperature Regime

The suitability curve for each year between 2003 and 2016 is shown in Figure 2C. In more than a decade (2003–2016), favorable time periods for fish (FMCC and Chinese sturgeon) spawning were from the middle of spring to early summer (April to June) and from the middle of autumn to early winter (October to December) based on the suitability index. Overall, in autumn, the temperature window for Chinese sturgeon spawning was delayed by 29 days. In spring, the suitable temperature windows for gonad development and spawning of the FMCC were delayed by 35 and 32 days, respectively (Figure 2C).

### 3.4. Future Trends

**Figure 3:**
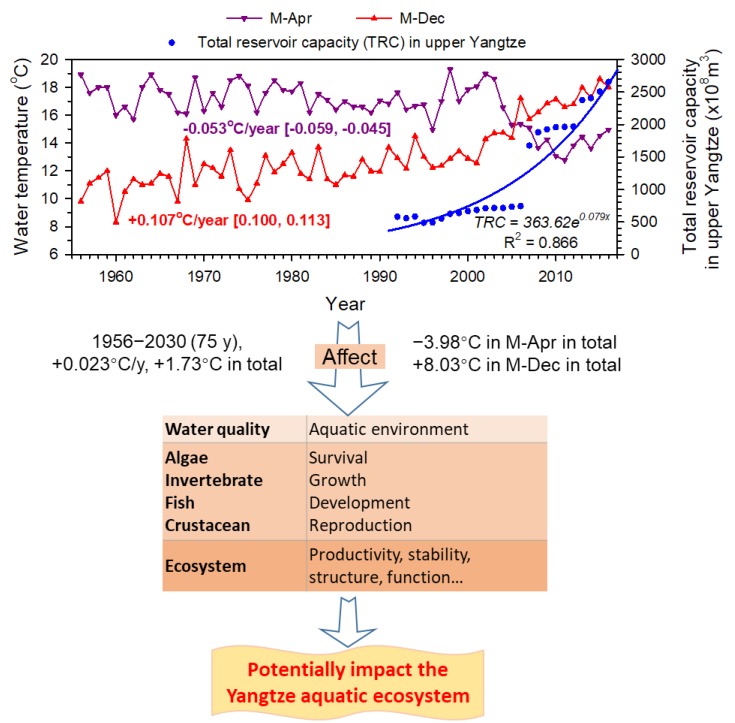
Increases in dam construction (water impounding) and increasing temperature shifts and how they potentially impact the whole Yangtze aquatic ecosystem. The equation between total reservoir capacity (TRC) and year (i.e., x) assumed x = 1 in 1992. According to the developing trends (Sen’s slopes), the cumulative alterations of water temperature in year, M-Apr and M-Dec during 1956–2030 are +1.73 °C, −3.98 °C, and +8.03 °C, respectively.

We found obvious trends of temperature alterations (Table 3, Figure 3). In M-Apr and M-Dec at Yichang during 1956–2016 (n = 61), Sen’s slopes were −0.053 °C/year (−0.059, −0.045) (*p*-value (Two-tailed) <0.0001) and +0.107 °C/year (0.100, 0.113) (*p*-value (Two-tailed) <0.0001) respectively, detected by Mann–Kendall trend tests (Figure 3, Supplemental Table S3). The reservoir capacity (i.e., dam construction and water impounding) made an obvious contribution to temperature alterations. Equations between water temperature in M-Apr (WT_M-Apr_) and M-Dec (WT_M-Dec_), and total reservoir capacity in the upper Yangtze (TRC_uY_) during 1992–2016 (n = 25) were: WT_M-Apr_ = 43.85 × TRC_uY_^–0.15^ (R^2^ = 0.60) and WT_M-Dec_ = 3.70 × TRC_uY_^0.20^ (R^2^ = 0.80), respectively. According to the developing trends (Sen’s slopes), from 2017 to 2030 the water temperature in M-Apr will continually decrease by approximately −0.74 °C, while in M-Dec it will unceasingly increase by about +1.50 °C. This implies further temperature regime shifts in the near future. In a long time-period during 1956–2030, the cumulative alterations of water temperature in a year, M-Apr and M-Dec, are +1.73 °C, −3.98 °C, and +8.03 °C, respectively.

## 4. Discussion

### 4.1. Limitations of the Temperature Profile Assessment Method

There were few limitations on research methods in this study. The temperature data used in this study were obtained over a long observation period from 1981 to 2016. In the earlier times, there was no strict standard procedure to measure river temperature. In China, the first standard procedure method was issued in 1991. The commonly used thermometers might not have calibrated accurately so that the accuracy of temperatures measured in early times might not be as accurate as that measured in present times. However, it would be reasonable to assume that a potential error occurred from temperature data from early times may not greatly affect the results of this study on the long-term trend changes. The hydrological monitoring station (Yichang) for understanding the temperature regimes has always been selected as the temperature reference point in the flow regimes in relation with Chinese sturgeon research. However, the species may have distributed at some distance from the monitoring station. Therefore, the temperature regime measured at the station might not be represented as distributed temperature of the species since the species might be freely locating [34]. It is assumed that this potential error would be very small and can be ignored in this study as the water temperature in a certain reach of the Yangtze main stem is normally very stable. The estimates on the gonad development status of the breeding population of the Chinese sturgeon were based on several captures at sampling sites. These would have had a few sampling variations due to gradual improvements (e.g., change in sampling grids) causing some early errors. In addition, heavy and selective capture might have slightly affected the structure of the cohorts/populations in the following year. This potential source of variability was ignored in this study since entire structures of the breeding population would not be seriously affected by those captures.

### 4.2. Gonad Development and Temperature Regime

Water temperature is important for the gonad development in sturgeons. Natural or artificial wintering (vernalization of 2–4 months at a lower temperature) is necessary before reaching the final maturation stage of breeders which is connected to successful spawning [27]. The complete controlled spawning of the Chinese sturgeon was accomplished by two institutes in 2009 and 2012, respectively [35,36]. The published results explained that the female breeders had been successfully cultured in the Three Gorges Reservoir or in land-based systems using the water from the reservoir because the water temperature regime used was very close to that of the historic spawning reach. Some other artificial environments (such as ponds or large-scale aquarium) had also been used to culture the breeders, but they did not develop well. These examples imply that a water temperature regime with a precise profile should be required to complete the maturation cycle of the breeder so that a full and functional gonad development can be achieved.

This study indicated that the high temperature might not necessarily cause a significant impact on the gonad development of Chinese sturgeon. However, note that the Yangtze River is a very large system with huge water volume and the water temperature may not be considerably high in the entire river during the summer. For example, in the historic spawning reach, the highest temperature recorded was approximately 23 °C. However, below the Gezhouba Dam that is the present remaining spawning area, the highest temperature was approximately 26 °C and in the downstream area far from the dam, the highest temperature was approximately 28–29 °C [37]. In the controlled water environment, if the water temperature was not controlled, the variation of temperature could be high and in summer the temperature in the controlled location can be extremely high. It was observed that a well-developing breeder degenerated after having been cultured in an outside pond during summer, where the water temperature reaches to higher than 30 °C. Therefore, it can be assumed that an upper (yet unknown) threshold and high temperatures may have an adverse impact on the gonad development and final maturation of the Chinese sturgeon.

### 4.3. Spawning Timing and Temperature Regime

The altered temperature regime may have various adverse effects on spawning timing. The timing of the spawning event can be related with hatching eggs, as the fish would prefer a favorable environment for the deposited eggs to be successfully hatched. At Yichang, the favorable temperature windows for spawning of the Chinese sturgeon were delayed year by year (Figure 2C). In comparison of temperature profiles between Yichang and historic spawning reaches, in recent years, this temperature window shifted by approximately 30–40 days. At Yichang, the suitable spawning temperature (<20 °C) started to be formed from M-Nov (Figure 2C). It was speculated that the fish could have a suitable spawning temperature earlier if they would be able to move downstream to spawn at the reach far from the Gezhouba Dam. In the Pearl River, the Chinese sturgeon is regarded to be spawning during the spring. However, in the Yangtze River, there was no indication that the fish could spawn during this season. However, there are suitable spawning temperatures from middle spring to early summer (M-Apr to M-Jun). Thus, theoretically the fish can spawn in the spring in the Yangtze River. In addition, other than water temperature, the photoperiod controls the endogenous readiness of female shortnose sturgeon *A. brevirostrum* to spawn [38]. This phenomenon on the combined timing of photoperiod change and temperature profile is also well known for teleosts to determine the seasonal onset of final maturation. However, any effect on a combination of a temperature profile and a photoperiod of spawning timing of the Chinese sturgeon is not studied. If the photoperiod is important for the gonad development and spawning timing of Chinese sturgeon, the delayed spawning temperature window could probably lead to a mismatch in the normal photoperiod time window. As a result, serious adverse effects on the reproductive cycle of breeders can be out of their phase of spawning.

### 4.4. Spawning Activity in 2013−2016 and Water Temperature

The spawning activities of Chinese sturgeon were related with the alterations of water temperature regime. In 2013, no spawning activity was observed in the spawning area below the Gezhouba Dam which had been used by the remaining sturgeon spawners for more than 30 years. According to the later investigation on the sturgeon juveniles in the middle and lower reaches as well as the estuary of Yangtze River, no juvenile was found. It was concluded that the spawning activity was completely interrupted in 2013 [20]. In 2014, no spawning activity was observed in the present spawning ground. However, in the later survey in 2015, more than 3000 juveniles in the Yangtze estuary were caught [39,40]. This means that there must have been unknown spawning activity somewhere in 2014. In addition, the number of juveniles was abundant and regarded to be the highest in recent years. Therefore, there must be one or more so far unknown spawning areas, although it is difficult to pinpoint where these new spawning sites are since the middle and lower reaches of the Yangtze River are extremely long. In 2015, no spawning activity was found in the whole river, while in 2016, a very small spawning activity was documented below the Gezhouba Dam [41]. It can be seen that during 2013–2015, the date reach to favorable spawning windows was obviously later than that of the previous years (except in 2006) (Figure 2C), that is probably the reason why the spawning activity was interrupted. However, in 2016, a small spawning took place, although the spawning window was also very late. It was speculated that the effects of water temperature on spawning (both gonad development and spawning timing) was complicated but delayed the spawning temperature window, which should be an adverse environmental factor.

However, as most animals search actively for favorable life conditions, the remaining spawners must have found an alternative mode of reproduction. This may be explained by an instinctive pattern of behavior that has developed during the evolution of the species [42]. Therefore, it can be speculated that locations of so far unknown new spawning areas were selected in accordance with combined conditions such as, (1) favorable temperature, (2) suitable riverbed morphology [14], and (3) preferable riverbed substrate [15]. Among these elements, water temperature is comparatively easy to monitor, thereby it can be used as an initial key element for research on further potential triggering factors relating to perceived new spawning areas.

### 4.5. Implications for Future River Management

The river temperature shifts will threaten the whole Yangtze aquatic ecosystem. In the 1980s, dam construction on a tributary (Ganjiang River) in the Yangtze Basin decreased the water temperature by 4–8 °C in the spawning season of Reeves shad *Tenualosa reevesii*. This was regarded as one of the critical reasons for the spawning failure and later extinction of the species [8,22]. There is no doubt that the altered river temperature will affect many aspects of the river ecosystem from the physical and chemical environment to aquatic organism and the whole ecosystem [1,2,3] (Figure 3). Currently, the cascade dam construction plan appears to be inevitable and only major remedial measures to water temperature (the multi-level water intake method) have limited effects [43] (Table 4).

Currently, the temperature regime in the Yangtze River (especially the shifts in the annual profile) has been drastically altered. Under natural conditions, the temperature regime shows some variations at various time-scales though [44]. However, due to hydro-dam construction, subsequent alterations of retention times (flow disruptions), and any influence by global warming, the alterations of the temperature regime have become much more obvious than before. In the Jinsha River and upper Yangtze reaches, 27 huge dams will be constructed in the near future [5], and a huge impact on the temperature regime in the downstream reaches is predicted. These changes may not only affect the reproductive mode of the Chinese sturgeon but also influence the reproductive capacities of many other commercially and ecologically important Yangtze fish species, such as four major Chinese carps. Furthermore, negative impacts on the entire aquatic ecosystem can be expected. In general, the major mitigation measures to regulate the temperature regime during sensitive periods could utilize the stratified water temperature profile in the created reservoirs, by feeding the downstream stretches with waters from a selected reservoir depth which exhibits the required temperatures. In fact, this strategy has been widely used elsewhere and proven as a useful method. However, the situation in the Yangtze River is very complicated, when considering at a large-scale with many cascading situations with huge water volumes. It requires specific research to solve the management problems to address the operational requirements for hydropower generation and the ecologically required river flow and temperature profile. In any case, regulating the temperature regime should be a very important task for research and management to develop strategies that meet the ecological requirements for the entire river basin ecosystem and the effective utility of the important water resource system. In this context, the Chinese sturgeon can be considered a role model species to optimize water resource management in the Yangtze River.

## 5. Conclusions

There is no doubt that temperature alterations in the upper Yangtze will coincide with the thermal effects of the Three Gorges Reservoir, and have cumulative effects on water temperature below the Gezhouba Dam: this will create a further shift in the thermal regime. We strongly emphasize the need to highlight the water temperature issue in the Yangtze River. Some fundamental protection measures [45,46,47], such as re-evaluating dam construction plans at a basin scale [46], or removing small dams on tributaries to create natural temperature refuges, should be considered. Otherwise, the increasing temperature shifts will threaten or even imperil the entire Yangtze aquatic ecosystem.

## Figures and Tables

**Table 1:** Indicators defined to describe the temperature regime in the Yangtze River during the observational periods. Time for examining the temperature was from November 1 to October 31 of the following year, which is the reproduction cycle of *Acipenser sinensis*.

Indicator Group	Temperature Regime Indicator	Reason for Indicator Definition
(1) Magnitude of monthly water temperature amplitude	Mean temperature for each calendar month, from November to October, subtotal 12 parameters	The adults need about one year to reach maturity, which month is important as trigger is unknown.
(2) High temperature	Annual maximum temperature: 1-day mean, 3-day means, 7-day means, 30-day means, 90-day means	To see whether the high temperature and duration of that are related with gonad development.
(3) Low temperature	Annual minimum temperature: 1-day mean, 3-day means, 7-day means, 30-day means, 90-day means	To test whether the low temperature and duration of that are related to gonad development.
(4) Cumulative temperature	The sum of temperature from November 1 to October 31 of the following year	To see the cumulative effects of temperature on gonad development.
(5) Timing of extreme temperature	Days of 1-day maximum and 1-day minimum from November 1 to October 31	To see whether the occurrence time of extreme temperature is related to gonad development.
(6) Relative temperature difference	Base temperature index: 7-day minimum temperature/mean temperature of the year	To see the effects of relative temperature difference on gonad development. This definition refers to the concept of Base flow in IHA (Indicators of Hydrologic Alteration).

**Table 2:** Temperature regime indicators and their correlations with maturation percentage (Correlation *_M_*) and degeneration percentage (Correlation *_D_*) of breeding population of *Acipenser sinensis* below the Gezhouba Dam. The present temperature regime indicators after the full operation of the Three Gorges Dam during 2010–2016 are also indicated. A temperature year was defined from November 1 to October 31 of the following year based on the reproduction cycle of *Acipenser sinensis*.

Indicator Group	Indicator	1982–2001, 2003 ^a^ (n = 21)				1984–1993 ^b^ (n = 10)				2010–2016 (n = 7)		
		Min	Max	Mean ± S.D.	Correlation *_M_* (*p*, 2-Tailed)	Min	Max	Mean ± S.D.	Correlation *_D_* (*p*, 2-Tailed)	Min	Max	Mean ± S.D.
(1) Magnitude of monthly water temperature	Nov Mean	15.10	17.52	16.60 ± 0.65	0.429 (0.053)	15.60	16.96	16.33 ± 0.49	0.298 (0.403)	18.91	20.79	19.85 ± 0.61
	Dec Mean	11.41	14.16	12.60 ± 0.79	0.602 ** (0.004)	11.49	13.27	12.38 ± 0.65	−0.288 (0.419)	16.62	18.47	17.27 ± 0.71
	Jan Mean	8.51	11.90	10.07 ± 0.82	0.528 * (0.014)	8.51	10.76	9.79 ± 0.73	−0.441 (0.202)	13.67	16.08	14.64 ± 0.87
	Feb Mean	8.02	11.75	10.21 ± 0.93	0.580 ** (0.006)	8.02	11.23	9.85 ± 0.96	−0.605 (0.064)	11.44	13.49	12.47 ± 0.74
	Mar Mean	10.77	14.50	12.66 ± 0.97	0.514 * (0.017)	10.77	13.82	12.29 ± 1.04	−0.595 (0.069)	10.59	12.79	11.93 ± 0.80
	Apr Mean	15.69	18.80	17.09 ± 0.68	0.362 (0.107)	16.33	17.54	17.00 ± 0.43	−0.468 (0.172)	12.93	14.85	14.02 ± 0.72
	May Mean	19.96	22.73	21.43 ± 0.70	0.205 (0.373)	19.96	22.16	21.20 ± 0.67	0.186 (0.607)	17.21	20.03	18.64 ± 1.08
	Jun Mean	22.65	24.47	23.55 ± 0.56	0.326 (0.149)	22.65	24.47	23.73 ± 0.65	−0.591 (0.072)	21.51	23.53	22.45 ± 0.79
	Jul Mean	23.45	26.43	24.57 ± 0.88	0.158 (0.494)	23.53	26.43	24.66 ± 0.87	−0.001 (0.998)	23.31	26.25	24.61 ± 1.06
	Aug Mean	24.14	28.70	25.73 ± 1.05	0.348 (0.123)	24.14	26.43	25.43 ± 0.84	0.285 (0.425)	24.69	26.54	25.84 ± 0.67
	Sep Mean	22.06	24.64	23.16 ± 0.78	0.381 (0.088)	22.06	24.23	22.80 ± 0.65	−0.411 (0.239)	23.15	26.16	24.82 ± 1.11
	Oct Mean	18.85	21.52	19.95 ± 0.77	0.470 * (0.031)	18.85	20.00	19.33 ± 0.40	−0.628 (0.052)	21.39	23.24	22.31 ± 0.65
(2) High temperature	1-day Max	25.80	29.70	27.28 ± 1.03	0.302 (0.196)	26.20	28.20	27.10 ± 0.76	−0.217 (0.547)	26.00	27.40	26.84 ± 0.53
	3-day Max	25.57	29.50	27.07 ± 1.00	0.275 (0.241)	25.87	28.07	26.88 ± 0.79	−0.155 (0.669)	25.93	27.33	26.80 ± 0.52
	7-day Max	25.29	29.34	26.86 ± 1.03	0.294 (0.208)	25.46	27.87	26.61 ± 0.83	−0.090 (0.805)	25.87	27.17	26.70 ± 0.51
	30-day Max	24.56	28.82	26.09 ± 1.00	0.307 (0.188)	24.56	26.79	25.77 ± 0.73	0.106 (0.770)	25.30	26.76	26.13 ± 0.60
	90-day Max	24.07	26.76	24.89 ± 0.62	0.320 (0.169)	24.07	25.50	24.76 ± 0.44	−0.027 (0.940)	23.94	26.15	25.16 ± 0.81
(3) Low temperature	1-day Min	7.40	10.90	9.05 ± 0.96	0.584 ** (0.007)	7.40	10.30	8.67 ± 0.90	−0.499 (0.142)	10.20	12.50	11.37 ± 0.89
	3-day Min	7.53	10.97	9.15 ± 0.94	0.568 ** (0.009)	7.53	10.40	8.79 ± 0.88	−0.478 (0.162)	10.20	12.50	11.44 ± 0.88
	7-day Min	7.64	11.04	9.29 ± 0.92	0.559 * (0.010)	7.64	10.56	8.95 ± 0.87	−0.475 (0.166)	10.29	12.53	11.55 ± 0.85
	30-day Min	7.88	11.55	9.67 ± 0.93	0.575 ** (0.008)	7.88	10.76	9.32 ± 0.87	−0.531 (0.114)	10.58	12.78	11.78 ± 0.79
	90-day Min	9.26	12.09	10.69 ± 0.75	0.670 ** (0.001)	9.26	11.21	10.31 ± 0.63	−0.740 * (0.014)	11.41	13.58	12.64 ± 0.76
(4) Cumulative temperature	Sum from Nov 1 to Oct 31	6392.7	6844.6	6637.7 ± 145.8	0.830 ** (0.000)	6446.7	6672.9	6551.7 ± 77.7	−0.891 ** (0.001)	6782.7	7250.6	6980.0 ± 158.9
(5) Timing of extreme temperature	Days of 1-day max to Oct 31	62	128	85.55 ± 16.69	0.348 (0.132)	62	122	83.20 ± 17.43	−0.364 (0.301)	48	86	66.14 ± 12.67
	Days of 1-day min to Oct 31	249	289	273.55 ± 13.08	0.312 (0.181)	249	289	271.30 ± 16.75	−0.364 (0.301)	228	253	239.00 ± 8.94
(6) Relative temperature difference	7-day min/year mean	0.43	0.59	0.51 ± 0.04	0.459 * (0.042)	0.43	0.59	0.50 ± 0.05	−0.398 (0.254)	0.55	0.64	0.60 ± 0.03

^a^ The scientific capture was interrupted in 2002, the maturation percentage of *Acipenser sinensis* was from Reference [26]. ^b^ The degeneration percentage of *Acipenser sinensis* was from Reference [11]. * Correlation is significant at the 0.05 level (2-tailed). ** Correlation is significant at the 0.01 level (2-tailed).

**Table 3:** Trend analysis of water temperature at Yichang station (river km 1669.2) during 1956–2016 (n = 61). The Yichang station is approximately 6 km below the Gezhouba Dam (Figure 1), the lowermost dam on the Yangtze main stem. The Three Gorges Dam is about 40 km upstream of the Gezhouba Dam. River km 0 is at the Yangtze estuary. The only known spawning area of the Chinese sturgeon was reported to be immediately below the Gezhouba Dam (i.e., about 5 km upstream of Yichang station) [14].

Season	Month	Kendall’s Tau	Trend	*p*-Value (Two-Tailed)	Sen’s Slope (95% Confidence Interval)
Spring	March	−0.201	Yes	0.022	−0.017 (−0.021, −0.013)
	April	−0.414	Yes	<0.0001	−0.050 (−0.055, −0.045)
	May	−0.203	Yes	0.021	−0.019 (−0.022, −0.014)
Summer	June	−0.146	No	0.099	−0.009 (−0.011, −0.006)
	July	−0.095	No	0.282	−0.007 (−0.010, −0.004)
	August	0.071	No	0.426	0.005 (0.001, 0.008)
Autumn	September	0.284	Yes	0.001	0.027 (0.022, 0.031)
	October	0.497	Yes	<0.0001	0.048 (0.044, 0.053)
	November	0.638	Yes	<0.0001	0.068 (0.063, 0.072)
Winter	December	0.670	Yes	<0.0001	0.103 (0.096, 0.110)
	January	0.701	Yes	<0.0001	0.086 (0.080, 0.093)
	February	0.506	Yes	<0.0001	0.051 (0.047, 0.055)
Year		0.548	Yes	<0.0001	0.023 (0.022, 0.024)

**Table 4:** Estimated river temperature alterations in 2023 and thereafter in the upper Yangtze when two large dams (Wudongde and Baihetan) are finished. It is assumed that the remedial water temperature regulation method (multi-level water intake facilities) had been adopted on Wudongde, Baihetan, and Xiluodu dams (but not on Xiangjiaba Dam, which was constructed without such facilities) (Figure 1) [43].

Season	Month	Rkm 2741.7 (Below Xiangjiaba Dam)	Rkm 2474.2 (Zhutuo, about 100 km to the Tail of Three Gorges Reservoir)
Spring	March	−0.2	0.8
	April	−3.3	−1.2
	May	−3.7	−1.8
Summer	June	−2.9	−1.7
	July	−1.4	−1.1
	August	−1.0	−1.2
Autumn	September	0.6	−0.1
	October	1.3	0.2
	November	3.5	1.1
Winter	December	5.3	2.6
	January	5.5	3.8
	February	2.9	3.6
Year		0.55	0.42

## Supplementary Materials

The following are available online at https://www.mdpi.com/2076-2615/9/8/583/s1, Table S1: Water temperature at Yichang hydrological monitoring station during 1981–2016 (°C), Table S2: Water temperature at four hydrological stations along the middle and lower Yangtze River during 1995–2014, in middle-April and middle-December (°C), Table S3: Water temperature at Yichang hydrological monitoring station during 1956–2016, in middle-April and middle-December (°C).
