# Supplementary figures and images for: Increasing River Temperature Shifts Impact the Yangtze Ecosystem: Evidence from the Endangered Chinese Sturgeon
Source: Animals (Basel). 2019 Aug 20;9(8):583. doi: 10.3390/ani9080583 (PMC6720547; doi:10.3390/ani9080583)

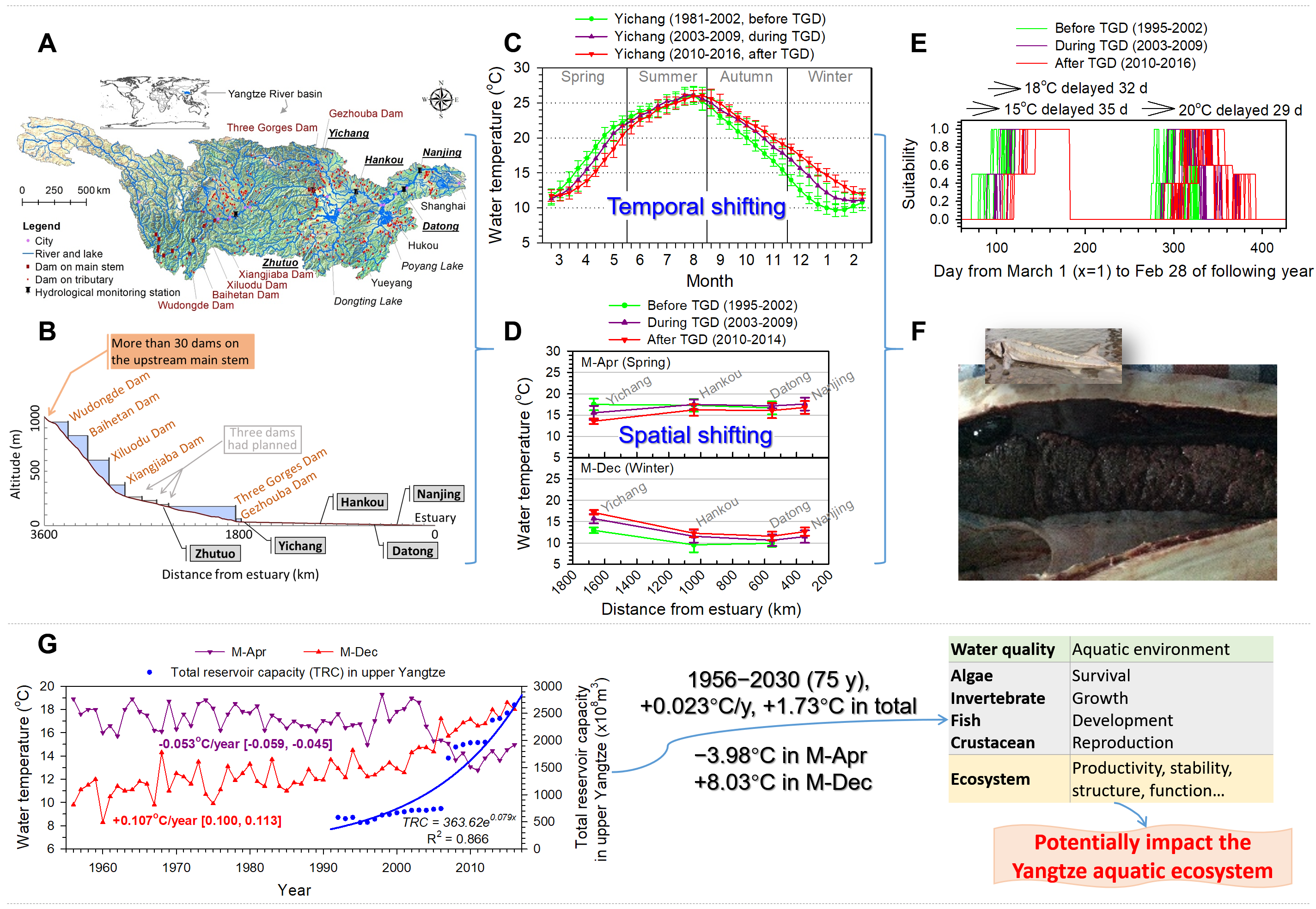

Supplement: Supplementary file 1 [file animals-09-00583-s001.zip › animals-561735-graphical.png]
